# Impact of Cannabis, Cannabinoids, and Endocannabinoids in the Lungs

**DOI:** 10.3389/fphar.2016.00317

**Published:** 2016-09-15

**Authors:** Caroline Turcotte, Marie-Renée Blanchet, Michel Laviolette, Nicolas Flamand

**Affiliations:** Centre de Recherche de l’Institut Universitaire de Cardiologie et de Pneumologie de Québec, Département de Médecine, Faculté de Médecine, Université Laval, Québec City, QCCanada

**Keywords:** cannabinoid, endocannabinoid, lung, inflammation, host defense

## Abstract

Since the identification of cannabinoid receptors in the 1990s, a research field has been dedicated to exploring the role of the cannabinoid system in immunity and the inflammatory response in human tissues and animal models. Although the cannabinoid system is present and crucial in many human tissues, studying the impact of cannabinoids on the lungs is particularly relevant because of their contact with exogenous cannabinoids in the context of marijuana consumption. In the past two decades, the scientific community has gathered a large body of evidence supporting that the activation of the cannabinoid system alleviates pain and reduces inflammation. In the context of lung inflammation, exogenous and endogenous cannabinoids have shown therapeutic potential because of their inhibitory effects on immune cell recruitment and functions. On the other hand, cannabinoids were shown to be deleterious to lung function and to impact respiratory pathogen clearance. In this review, we present the existing data on the regulation of lung immunity and inflammation by phytocannabinoids, synthetic cannabinoids and endocannabinoids.

## Impact of Cannabis and Its Constituents On Lung Functions

Numerous countries have been considering decriminalizing and/or legalizing cannabis use. In that regard, it has become crucial for health agencies to gain a better understanding of how cannabis and its constituents (cannabinoids) impact humans. This article reviews the impact of cannabis consumption on the lungs as well as the impact of the endocannabinoid system in the regulation of lung functions, given that the most popular route of cannabis ingestion is smoking.

The impact of marijuana smoking on structural and immune cells of the lungs has been of interest to researchers for several decades. In 1985, a histological examination of lung tissue sections was done on known marijuana smokers who had died suddenly (Morris, 1985). The author found macrophage infiltrates in the lungs of marijuana smokers, which were often accompanied by fibrosis with lymphocytes in areas filled with macrophages. Considering the circumstances in which the study was conducted, it was not possible to obtain detailed information about marijuana and tobacco use by these individuals, making it complex to confirm that these cellular changes were caused by marijuana alone. It is now known that cannabis induces effects on lung functions that are not found in tobacco smokers. Although there are marked differences in the composition of marijuana smoke versus tobacco smoke (Moir et al., 2008), studies that include groups of non-smokers, marijuana smokers, and tobacco smokers can provide good insight into the role of marijuana and its constituents on the structures and functions of the lungs (Owen et al., 2014).

Many studies have described lung damage in marijuana smokers, as well as cellular damages in cells exposed to marijuana smoke or cannabinoids *in cellulo*. Gong et al. (1987) performed bronchial biopsies on volunteers and found airway hyperemia in both marijuana- and tobacco-smoking groups, as opposed to non-smokers. The authors also observed hyperplasia of goblet and basal cells in both marijuana and tobacco smokers, with a tendency toward higher hyperplasia in marijuana smokers. Of note, cellular disorganization was present in over 50% of marijuana smokers, but was not found in tobacco smokers. However, this type of experimental design still does not provide defined conditions in terms of exposure to cannabis and/or tobacco. In this respect, a study was performed on primates that were exposed to a low or high dose of marijuana, placebo cigarettes or sham smoke (Fligiel et al., 1991). Cannabis exposures were defined as two marijuana cigarettes per week for the low-dose group, and seven marijuana cigarettes per week for the high-dose group. Epithelial hyperplasia was found in all groups, but there was a greater incidence and severity of bronchiolitis, alveolar cell hyperplasia with atypia and fibrosis in marijuana smokers. These findings indicate that cannabis induces some types of cellular damage to the lungs that are not observed with tobacco smoke. Therefore, some bioactive constituents of marijuana (cannabinoids) might be responsible for these effects, suggesting that the endocannabinoid system could also play a role in the regulation of pulmonary health and diseases.

### The Cannabinoid Receptors and the Lungs

The endocannabinoid system comprises cannabinoid receptors (CB_1_ and CB_2_), their endogenous ligands (endocannabinoids) and the enzymes involved in their metabolism. Some cannabinoids, such as (–)-*trans*- Δ^9^-tetrahydrocannabinol (Δ^9^-THC), can activate the cannabinoid receptors (Felder et al., 1995). Others, such as cannabinol and cannabidiol, modulate cell functions through other means (Russo et al., 2005; Kathmann et al., 2006; Ryberg et al., 2007). The two best characterized endocannabinoids, namely 2-arachidonoyl-glycerol (2-AG) and arachidonylethanolamide (AEA) also activate cannabinoid receptors. In addition, endocannabinoids activate additional receptors and can be metabolized into other bioactive compounds that can modulate cell functions (Turcotte et al., 2015). In the lungs, the cannabinoid receptors were found on structural cells and most leukocytes.

A study of the distribution of cannabinoid receptors among human organs found both CB_1_ and CB_2_ mRNA in the lungs and the bronchial tissue, with CB_1_ mRNA levels being significantly higher than those of CB_2_ (Galiegue et al., 1995; Papadaki, 2013). To this date, there has been no report of the expression of these receptors in primary airway epithelial cells specifically, although the mRNA and protein for both receptors were detected in the 16HBE14o^-^ cell line (Gkoumassi et al., 2007). Given that airway epithelial cells actively participate in the recognition of pathogens as well as the recruitment of leukocytes in inflammatory conditions (Weitnauer et al., 2016), their ability to respond to cannabinoids and endocannabinoids should be further investigated. To our knowledge, there has not been any report of the expression of cannabinoid receptors by human lung fibroblasts.

Among immune cells residing in the airways, alveolar macrophages (AMs) are by far the most numerous, constituting over 90% of the cells recovered by bronchoalveolar lavage (Hunninghake et al., 1979). A recent study was performed on human lung-resident macrophages that were isolated from the lung tissue of patients with adenocarcinoma (Staiano et al., 2016). CB_1_ and CB_2_ mRNA and proteins were detected in both tumor-associated and non-tumor macrophages. CB_2_ levels were higher than those of CB_1_, a pattern that the authors also observed in monocyte-derived macrophages. Interestingly, the cannabinoid receptors expressed in lung macrophages were functional in contrast to those of monocyte-derived macrophages, as assessed by extracellular signal-regulated kinases 1/2 (ERK1/2) phosphorylation assays. This emphasizes the difference between tissue-resident and blood-derived macrophages, as well as the importance of tissue-derived factors in the modulation of macrophage functions. Although they are the most numerous in alveoli, other leukocytes involved in lung immunity also express cannabinoid receptors. Human monocyte-derived dendritic cells (DCs) express both the CB_1_ and CB_2_ receptors, and they also have the ability to synthesize AEA and 2-AG (Matias et al., 2002). Both CB receptors were also found in murine bone marrow-derived DCs (Do et al., 2004; Lu et al., 2006b).

The presence of cannabinoid receptors on circulating leukocytes can also impact lung immunity, as many of these cells are recruited to the lungs at different stages of the inflammatory response. Eosinophils, for example, express large amounts of CB_2_ receptors (Oka et al., 2004; Chouinard et al., 2011, 2013). Monocytes express both cannabinoid receptors, with a predominant expression of CB_2_ (Galiegue et al., 1995; Montecucco et al., 2008; Han et al., 2009; Castaneda et al., 2013). It is less clear whether or not neutrophils express CB_2_, since discrepancies regarding its expression have been reported (Galiegue et al., 1995; Kurihara et al., 2006; Chouinard et al., 2013). A more detailed summary of cannabinoid receptor expression by human leukocytes has recently been reviewed (Turcotte et al., 2015). The role of cannabinoid receptors in modulating the effector functions of these leukocytes is discussed in the next sections.

### Impact of Cannabis and Cannabinoids on Structural and Inflammatory Cells Found in the Lungs

A limited number of studies investigated the impact of cannabinoids on structural cells found in the lungs. However, Δ^9^-THC changes the expression pattern of numerous genes in primary human airway epithelial cells, notably *PTGS2* and *IL1A*, which encode cyclooxygenase-2 and the proinflammatory cytokine interleukin (IL)-1α, respectively. Moreover, it causes a decrease in cell viability and mitochondrial membrane potential in these cells (Sarafian et al., 2005). Δ^9^-THC also caused mitochondrial damage in the lung epithelial cell line A549 (Sarafian et al., 2003). The use of A549 cells overexpressing the CB_2_ receptor indicated that some of the effects of Δ^9^-THC on the airway epithelium are CB_2_-dependent (Sarafian et al., 2008). As for lung fibroblasts, early *in vitro* studies reported that cannabinoids can stimulate them to synthesize prostaglandin E_2_ (Burstein et al., 1983, 1986). However, these experiments were performed in the WI-38 cell line and the involvement of cannabinoid receptors was not elucidated, since these studies were published before they were cloned. To our knowledge, these findings have not been confirmed in primary lung fibroblasts, and the involvement of CB receptors has yet to be determined.

The first line of defense in the lungs against aerosolized bacteria, viruses and toxins consists of resident cells such as bronchial epithelial cells, AMs and DCs. When they encounter a pathogen, they are able recognize it through receptors binding specific molecular motifs found on microorganisms. This triggers signaling events leading to the transcription of pro-inflammatory genes and the release of mediators that induce the recruitment of leukocytes from the circulation. In response to these chemotactic factors, neutrophils are the first cells to be recruited to the lungs. They exert key effector functions and thus, participate in pathogen elimination. Lung homeostasis is restored with the help of AMs that have acquired a pro-resolution, wound-healing phenotype.

Numerous *in cellulo* studies assessed the impact of cannabinoids on human and murine macrophage functions. Experiments performed with mouse peritoneal macrophages demonstrated that Δ^9^-THC downregulates NO (nitric oxide) production in these cells, as well as tumor necrosis factor (TNF)-α maturation and secretion (Coffey et al., 1996; Zheng and Specter, 1996). In RAW264.7 macrophages, the impact of Δ^9^-THC on lipopolysaccharide (LPS)-induced NO production was shown to be a consequence of NF-κB inhibition (Jeon et al., 1996). The impact of Δ^9^-THC was also investigated in the P388D1 cell line and was shown to impair phagocytic activity of these murine macrophages (Tang et al., 1992).

In light of these findings, some have studied the functions of human AMs isolated from healthy volunteers and marijuana smokers and these reports are consistent with the data obtained from murine macrophages. AMs from marijuana smokers were found to have a decreased capability to ingest/kill *Staphylococcus aureus* (Baldwin et al., 1997) and produced less NO following LPS stimulation (Shay et al., 2003). This was not the case in AMs recovered from tobacco smokers, indicating that these effects were unlikely to be caused by the smoke itself. The incubation of these AMs with GM-CSF or interferon (IFN)-γ restored their ability to produce NO, suggesting that marijuana exposure causes a decrease in cytokine priming that weakens host defense (Roth et al., 2004).

Macrophages and DCs are highly potent antigen presenting cells (Kopf et al., 2015). DCs constitute a crucial bridge between innate and adaptative immunity. The immature, lung-resident DCs that are located close to the epithelial surface of the respiratory tract can capture and process antigens that enter the lungs. They subsequently migrate to the lymph nodes, where they present the antigen to naïve T cells. Given that they both express cannabinoid receptors, AMs and DCs can respond to exogenous cannabinoids or endocannabinoids, thereby modulating immune responses. In murine cells, Δ^9^-THC was shown to impair LPS-induced bone marrow-derived DC maturation (Karmaus et al., 2013a). Moreover, treatment of these DCs with Δ^9^-THC before cultivating them with CD8+ cells reduced their ability to produce IFN-γ, an effect that was dependent on cannabinoid receptors. The CB_2_ receptor was later implicated in the modulation of DC functions, in a study showing that CB_2_ receptor agonists decreased DC migration by reducing matrix metalloproteinase (MMP)-9 production (Adhikary et al., 2012). This cannabinoid-induced decrease in DC migration occurred both *in vivo* and *in vitro*. In *Legionella pneumophila*-infected DCs, Δ^9^-THC treatment impairs Th1 cytokine production, in addition to downregulating the expression of costimulatory molecules as well as major histocompatibility complex II (Lu et al., 2006a). This effect was later found to involve both cannabinoid receptors (Lu et al., 2006b). Altogether, these studies point to a detrimental effect of cannabinoids, particularly Δ^9^-THC, on antigen presentation, which impairs the immune response to pulmonary pathogens.

## The Endocannabinoid System and the Lungs

Endocannabinoids are known to regulate immune cell functions, either through cannabinoid receptors or through numerous metabolites (Turcotte et al., 2015). As discussed in Section “The Cannabinoid Receptors and the Lungs,” a growing body of evidence indicates that the endocannabinoid system is present in human lungs, with most cell types expressing cannabinoid receptors. Moreover, many structural and immune cells have the ability to synthesize endocannabinoids when exposed to inflammatory stimuli. The impact that this can have on lung homeostasis and disease is not fully understood, and this section discusses the existing *in cellulo* and *in vivo* evidence.

### Impact of Endocannabinoids on Structural Cells

Although airway epithelial cells constitute an extremely large contact surface and an important physical barrier, very few studies have investigated the impact of endocannabinoids on their functions, with reports often focusing on immune cells. A recently published study demonstrates that the endocannabinoid AEA increases permeability of the airway epithelial cell line Calu-3 (Shang et al., 2016). Of note, this effect was caused by arachidonic metabolites rather than cannabinoid receptor activation. This underscores that airway epithelial cells do not only respond to exogenous cannabinoids, but also have the ability to metabolize endocannabinoids into various bioactive lipid mediators that can modulate immune cell functions. In this respect, Nomura et al. showed that 2-AG is an important source of prostaglandins in the lungs of mice (Nomura et al., 2011). These findings underscore the importance of 2-AG as a source of arachidonic acid in the lungs, which can also be metabolized by human neutrophils and eosinophils into leukotriene B_4_ and C_4_, respectively (Chouinard et al., 2011; Larose et al., 2014).

### Impact on Immune Cells

#### Alveolar Macrophage Functions

To our knowledge, there is currently no evidence of the regulation of human AM functions by endocannabinoids. However, a limited number of studies investigated the impact of 2-AG and AEA on macrophage cell lines. In this regard, AEA, but not 2-AG, was found to impair NO and cytokine production in the J774 murine macrophage cell line (Chang et al., 2001). The potentiating effect of 2-AG on NO production was likely caused by its metabolites arachidonic acid and prostaglandin E_2_, as both lipids mimicked this effect. Moreover, there is evidence of J774 macrophages being capable of synthesizing PGD_2_-G and PGE_2_-G, which are COX-2 metabolites of 2-AG (Alhouayek et al., 2013). The exogenous addition of PGD_2_-G to J774 cultures downregulated LPS-induced IL-1β production by these cells, whereas the addition of PGE_2_-G or PGF_2α_-G had the opposite effect. Finally, PGD_2_-G reduced LPS-induced inflammation *in vivo*. These findings, in addition to a previous report showing that PGE_2_-G stimulated calcium mobilization in RAW267.4 cells (Nirodi et al., 2004), support that COX-2 metabolites of endocannabinoids regulate macrophage functions, although more research is prompted in order to identify the receptors that mediate these effects. Whether or not these mechanisms translate to human AMs and to lung inflammation also remains to be investigated.

#### Leukocyte Recruitment

Leukocyte recruitment to the lungs is a complex process that involves a cellular response to a chemotactic gradient and transmigration through the endothelium. The mechanisms of leukocyte chemotaxis modulation by cannabinoids varies from one cell type to another. Noteworthy, the endocannabinoid 2-AG was shown to exert effects on the endothelium itself, thus promoting Jurkat T cell adhesion and transmigration (Sarafian et al., 2008). These findings should be confirmed using primary human leukocytes in order to clarify the possible role of this mechanism in inflammatory disease.

Despite the conflicting data regarding the expression of CB receptors in neutrophils, the impact of various cannabinoids on neutrophil chemotaxis has been investigated. In one report, the pretreatment of human neutrophils with 2-AG or the CB_2_ agonist JWH015^1^ impaired their chemotactic response to the formylated peptide fMLP (Kurihara et al., 2006). The impact of 2-AG was blocked by the CB_2_ antagonist SR144528. Another study found that 2-AG and AEA do not induce neutrophil migration *in vitro* (Chouinard et al., 2011). However, 2-AG can stimulate other functions of human neutrophils, such as bioactive lipid synthesis, antimicrobial peptide release and oxidative burst (Chouinard et al., 2013). Importantly, these effects were shown to be CB_2_-independent, underscoring the importance of endocannabinoid metabolites in the regulation of neutrophil functions.

Endocannabinoids have also been implicated in eosinophil recruitment. Eosinophils are granulocytes that are found in minimal numbers in the blood of healthy individuals, but their levels are elevated in allergic diseases such as atopic dermatitis and asthma, or in parasitic infections. Their recruitment to the bronchial tissue and the airways is a hallmark of asthma and they actively participate in the onset and persistence of a chronic inflammatory state. As mentioned in Section “The Cannabinoid Receptors and the Lungs,” human eosinophils strongly express the CB_2_ receptor, making them highly responsive to cannabinoids. In this respect, 2-AG was found to be a chemoattractant for human primary eosinophils *in vitro* (Oka et al., 2004; Kishimoto et al., 2006; Larose et al., 2014). Noteworthy, the eosinophil differentiation factor and priming agent IL-5 greatly potentiates the effect of 2-AG (Larose et al., 2014). Furthermore, the CB_2_ receptor agonist JWH133 primed the response of eosinophils toward other chemoattractants (Frei et al., 2016). Although the principle of 2-AG-induced migration of eosinophils was confirmed *in vivo* in a murine model of dermatitis (Oka et al., 2006), the important impact that this could have on eosinophil migration to the lungs in asthmatic subjects remains to be defined. Of note, there have been two distinct case reports of individuals who developed an eosinophilic pneumonia following marijuana consumption (Liebling and Siu, 2013; Natarajan et al., 2013) suggesting that cannabinoids could promote and/or induce an excessive recruitment of eosinophils to the lungs in humans.

## Impact Of Cannabinoids On Host Defense *In Vivo*

### Evidence from Animal Models

Given how cannabinoids can modulate immune cell functions (as detailed in Section “Impact of Cannabinoids on Acute Lung Inflammation”), it is likely that they have an impact on host defense to various pathogens. In this respect, murine models of lung infection were used to assess the involvement of cannabinoids in immunity against viruses and bacteria. **Table 1** summarizes the data obtained from animal models of pulmonary infection. Noteworthy, the administration of cannabinoids in these protocols was never in the form of cannabis smoking, but rather as the direct administration of cannabinoids. These studies all point to the conclusion that the administration of plant-derived or synthetic cannabinoids impairs pathogen clearance and in certain cases, increases mortality. Of note, one study used cannabinoid receptor-deficient mice in their model of influenza infection, underscoring the involvement of cannabinoid receptors in the detrimental effects of Δ^9^-THC on lung immunity (Karmaus et al., 2011).

**Table 1:** Impact of cannabinoids on pulmonary host defense in mouse models and humans.

Treatment	Dose (administration route)	Model (species)	Effects	Reference
Δ^9^-THC	25–75 mg/kg (oral gavage)	Influenza A/PR/8/34 infection (mouse)	↑ Viral hemagglutinin 1 mRNA	Buchweitz et al., 2007
			↓ CD4+ and CD8+ cells in BALF	
			↓ Macrophages in BALF	
	75 mg/kg (oral gavage)		↓ IFN-γ-producing CD4+ cells	Karmaus et al., 2013a
			↓ IL-17-producing NK cells	
			↓ Antigen-presenting cells and inflammatory myeloid cells	
	
	8 mg/kg (i.v.)	*Legionella pneumophila* infection (mouse)	↑ Mortality	Klein et al., 1993; Smith et al., 1997
			↑ Bacterial load in lungs	
			↑ Serum levels of acute-phase cytokines	
			
			Shift from Th1 to Th2 immunity	Newton et al., 2009

CBN	6 mg/kg (i.v.)	*Legionella pneumophila* infection (mouse)	↑ Mortality	Smith et al., 1997

			↑ Bacterial load in lungs	
CBD	8-16 mg/kg (i.v.)	*Legionella pneumophila* infection (mouse)	No significant effects on mortality or bacterial load	Smith et al., 1997

CP55,940	6 mg/kg (i.v.)	*Legionella pneumophila* infection (mouse)	↑ Mortality	Smith et al., 1997

			↑ Bacterial load in lungs	
CB_1_^-/-^CB_2_^-/-^	n/a	Influenza A/PR/8/34 infection (mouse)	↑ Pro-inflammatory mediator mRNA	Karmaus et al., 2011
			↑ T cell activation	
			↑ IL-17 and IFN-γ production by NK cells and T cells	
			↑ APC maturation	

*BALF, bronchoalveolar lavage fluid; CBD, cannabidiol; CBN, cannabinol; NK, natural killer; APC, antigen presenting cells; i.v., intravenous.*

## Impact of Cannabinoids on Acute Lung Inflammation

Acute lung injury (ALI) is characterized by a disruption of the vascular endothelium and the alveolar epithelium. There are numerous causes for ALI, including sepsis, pneumonia, aspiration of gastric contents, toxic gas inhalation, trauma and blood transfusion. At the cellular level, ALI causes the loss of alveolar–capillary membrane integrity, excessive neutrophil transmigration, and the release of pro-inflammatory mediators (Johnson and Matthay, 2010). Endotoxins such as LPS are often used to induce acute lung inflammation in animals, as this results in a pulmonary edema and neutrophilia mimicking human ALI. The impact of endocannabinoids, cannabinoids and even synthetic CB receptor agonists has been studied in these animal models of ALI. **Table 2** summarizes the effects of cannabinoid treatment on the disease, which are, in most cases, beneficial. One study found that cannabidiol treatment worsens LPS-induced ALI (Karmaus et al., 2013b), contradicting previous studies that showed the opposite (**Table 2**). However, the route of administration of CBD was not consistent from one study to the other (oral gavage vs. intraperitoneal), possibly accounting for the different impact observed. Of note, a study by Costola-de-Souza et al. (2013) used the monoacylglycerol lipase inhibitor JZL184 to increase 2-AG levels in the lungs of ALI mice, which resulted in an attenuation of ALI. Of note, CB_1_ and CB_2_ receptor antagonists partially blocked the impact of JZL184, indicating that other mechanisms are possibly involved in the effect of JZL184. Given that JZL184 can prevent the 2-AG-induced LTB_4_ biosynthesis by neutrophils as well as prostaglandin synthesis in the murine lung (Chouinard et al., 2011; Nomura et al., 2011), it is possible that the effect of JZL184 implicates not only the activation of cannabinoid receptors but also a decrease in the synthesis of several eicosanoids. Nevertheless, the positive impact that this approach had on lung damage and inflammation underscores the potential of such pathway modulation strategies in ALI therapy development.

**Table 2:** Impact of cannabinoids on pulmonary inflammation.

Treatment	Dose (administration route)	Model (species)	Effects	Reference
**ANTI-INFLAMMATORY**		
Δ^9^-THC	0.83 mg/kg (i.n.)	LPS-induced lung inflammation (mouse)	↓ Neutrophils in BALF	Berdyshev et al., 1998
	
			↓ TNF-α	
	20 mg/kg (i.p.)	Endotoxin B-induced lung inflammation (mouse)	↓ Mortality	Rao et al., 2015
			↑ Airway resistance	
			↓ Vascular leak	
			↓ Leukocyte infiltration	
			↓ Pro-inflammatory cytokines	

WIN55,212-2	3.9 μg/kg (i.n.)	LPS-induced lung inflammation (mouse)	↓ Neutrophils in BALF	Berdyshev et al., 1998
			↓ TNF-α	

AEA	26.1 ng/kg (i.n.)	LPS-induced lung inflammation (mouse)	↓ Neutrophils in BALF	Berdyshev et al., 1998
			↓ TNF-α	

CBD	20-80 mg/kg (i.p.)	LPS-induced lung inflammation (mouse)	↑ Lung function	Ribeiro et al., 2012; Ribeiro et al., 2015
			↓ Leukocyte recruitment	
			↓ Pro-inflammatory cytokines	
			↓ Myeloperoxidase	

JWH133	1 mg/kg (i.p.)	Neurogenic pulmonary edema (rat)	↓ Mortality	Fujii et al., 2016
			↓ Myeloperoxidase	

	5–20 mg/kg (i.p.)	Paraquat-induced lung injury (rat)	↓ Pulmonary edema	Liu et al., 2014
			↓ Lung damage	
			↓ Neutrophil infiltration	

JZL184	16 mg/kg (i.p.)	LPS-induced acute lung injury (mouse)	↓ Leukocyte recruitment	Costola-de-Souza et al., 2013
			↓ Lung damage	
			↓ Pro-inflammatory cytokines	


**PRO-INFLAMMATORY**		

CBD	75 mg/kg (oral gavage)	LPS-induced lung inflammation (mouse)	↑ Inflammatory cell infiltration	Karmaus et al., 2013b
			↑ LPS-induced pulmonary lesions	
			↑ TNF-α, IL-6, IL-23 and G-CSF mRNA	

*G-CSF, granulocyte-colony stimulating factor; i.n., intranasal; i.p., intraperitoneal.*

## Conclusion and Perspectives

Initial studies unraveled that cannabis smoking has deleterious effects on lung function and inflammation, some of which are not caused by tobacco smoking. Of note, cannabinoids are involved in many of these effects. More recently, new tools and animal models indicate that the downregulation of immune cell functions by cannabinoids/endocananbinoids could dampen pulmonary inflammation to such an extent that it could diminish host defense. Thus, it becomes urgent to revisit the impact of cannabis smoking, cannabinoids and endocananbinoids on lung damages and pulmonary inflammation with more recent molecular and pharmacological tools. In addition, it is unclear to which extent the data obtained in animal models are translatable to humans. In that regard, additional studies should be undertaken to better decipher the impact of cannabis smoking and/or administration on human lung integrity and functions as well as to delineate if targeting endocannabinoids might provide new or complementary benefits compared to cannabinoids. Given that some of the anti-inflammatory effects of endocannabinoids are mediated by their metabolites, the modulation of endocannabinoid metabolic pathways might prove itself a better alternative to increase the beneficial effects of the cannabinoid system while limiting the detrimental ones.

## Author Contributions

All authors listed, have made substantial, direct and intellectual contribution to the work, and approved it for publication.

## Conflict of Interest Statement

The authors declare that the research was conducted in the absence of any commercial or financial relationships that could be construed as a potential conflict of interest.

## Footnotes

1 **Chemical names**: JWH015 = (2-Methyl-1-propyl-1H-indol-3-yl)-1-naphthalenylmethanone, CP55,940 = (–)-*Cis*-3-[2-hydroxy-4-(1,1-dimethylheptyl)phenyl]-*trans*-4-(3-hydroxypropyl)cyclohexanol, JWH133 = (6aR,10aR)-3-(1,1-dimethylbutyl)-6a,7,10,10a-tetra-hydro-6,6,9-trimethyl-6H-dibenzo[b,d]pyran, JZL184 = 4-[Bis(1,3-benzodioxol-5-yl)hydroxy-methyl]-1-piperidinecarboxylic acid 4-nitrophenyl ester, SR144528 = 5-(4-chloro-3-methyl-phenyl)-1-[(4-methylphenyl)methyl]-*N*-[(1S,2S,4R)-1,3,3-trimethylbicyclo[2.2.1]hept-2-yl]-1H-pyrazole-3-carboxamide, WIN55,212-2 = [(3R)-2,3-dihydro-5-methyl-3-(4-morpholinylmethyl) pyrrolo[1,2,3-de]-1,4-benzoxazin-6-yl]-1-naphthalenyl-methanone, monomethanesulfonate.
